# A drug use survey among clients of harm reduction sites across British Columbia, Canada, 2012

**DOI:** 10.1186/1477-7517-11-13

**Published:** 2014-04-27

**Authors:** Margot Kuo, Arash Shamsian, Despina Tzemis, Jane A Buxton

**Affiliations:** 1 British Columbia Centre for Disease Control, 655 West 12th Avenue, Vancouver, BC V5Z 4R4, Canada; 2 School of Population and Public Health, University of British Columbia, 2206 East Mall, Vancouver, BC V6T 1Z3, Canada

**Keywords:** Harm reduction, Persons who use drugs, Survey, Surveillance

## Abstract

**Background:**

In British Columbia (BC), understanding of high-risk drug use trends is largely based on survey and cohort study data from two major cities, which may not be representative of persons who use drugs in other regions. Harm reduction stakeholders, representing each of the five geographic health regions in BC, identified a need for data on drug use to inform local and regional harm reduction activities across the province. The aims of this project were to (1) develop a drug use survey that could be feasibly administered at harm reduction (HR) sites across all health regions and (2) assess the data for differences in reported drug use frequencies by region.

**Methods:**

A pilot survey focusing on current drug use was developed with stakeholders and administered among clients at 28 HR supply distribution sites across the province by existing staff and peers. Data were collated and analysed using univariate and bivariate descriptive statistics to assess differences in reported drug use frequencies by geography. A post-survey evaluation was conducted to assess acceptability and feasibility of the survey process for participating sites.

**Results:**

Crack cocaine, heroin, and morphine were the most frequently reported drugs with notable regional differences. Polysubstance use was common among respondents (70%) with one region having 81% polysubstance use. Respondents surveyed in or near their region's major centre were more likely to report having used crack cocaine (*p* < 0.0001) and heroin (*p* < 0.0001) in the past week as compared to those residing >50 km from the major centre. Participants accessing services >50 km from the regional centre were more likely to have used morphine (*p* < 0.0001). There was no difference in powder cocaine use by client/site proximity to the regional centre. Participating sites found the survey process acceptable, feasible to administer annually, and useful for responding to client needs.

**Conclusions:**

The survey was a feasible way for harm reduction sites across BC to obtain drug use data from clients who actively use drugs. Drug use frequencies differed substantially by region and community proximity to the regional centre, underlining the need for locally collected data to inform service planning.

## Background

Illicit drug use is a major contributor to premature mortality and preventable morbidity and disability worldwide [1]. In Canada, there is geographic variation in drug use often based on regional drug trafficking activities affecting drug availability [2,3]. In the past decade, there has been increasing prevalence of crack cocaine smoking and decreased use of heroin outside of major port cities with a concurrent increase in the use of diverted prescription opioids [4-6]. Changes in drug supply and method of use may put persons who use drugs (PWUD) at higher risk of overdose and other harms due to lack of familiarity with a new drug's potency and health risks [7].

In British Columbia (BC), there is monitoring of drug use trends and harms experienced among high-risk populations. Drug information is collected in the context of cohort studies or cross-sectional surveys in sentinel city sites. Long-running cohort studies include the Vancouver Injection Drug Use Study, which assesses health status of persons who inject drugs (PWID) in Vancouver [8]. The At-Risk Youth Study is an affiliated cohort study focusing on at-risk youth in Vancouver aged 14–26 years [9]. The Cedar Project is a prospective cohort study of young aboriginal persons in Vancouver and Prince George [10]. The High Risk Populations survey, conducted in Vancouver and Victoria by The Centre for Addictions Research (CARBC), provides semi-annual indicators of drug use patterns and harms experienced in three at-risk populations: recreational/party attendees, street-involved youth, and street-involved adults [11].

In Canada, there are two nationally coordinated cross-sectional surveys, I-track and M-track focusing on PWID and men who have sex with men, respectively [12]. This work provides valuable assessment of risk factors for communicable disease transmission and other harms.

The US Centers for Disease Control guidelines for evaluating public health surveillance systems outline seven key attributes: simplicity, flexibility, acceptability, sensitivity, positive predictive value, representativeness, and timeliness [13]. Fielden and Marsh reported that Canadian drug use systems are neither timely enough for response to emerging hazards nor representative of areas outside of major metropolitan centres [14]. In BC, stakeholders in the area of harm reduction identified a gap in knowledge regarding drug use patterns and harms experienced by PWUD outside the major cities of Vancouver and Victoria. Knowledge of drug use trends in different areas of the province is needed to optimize area-level harm reduction (HR) services and to inform public health responses to prevalent drug-related issues.

The provincial Harm Reduction Program is supported by the BC Ministry of Health and overseen by the BC Harm Reduction Strategies and Services (HRSS) Committee, with health authority HR coordinators from each of the five geographic health authorities, First Nations Health Authority, and BC Ministry of Health and managed by the BC Centre for Disease Control (BCCDC) [15]. The programme aims to reduce infectious diseases and other drug-related harms among PWUD and other vulnerable populations, such as survival sex workers, by distributing supplies for safer sex, injection, and inhalation practices. There is a network of over 200 HR sites that order supplies directly from the provincial programme in communities of each of the five geographic health authorities (range 15–110) with satellite sites obtaining supplies from the ordering sites [16]. HR sites may be located at public health units or community service organizations, and HR stakeholders may include public health practitioners, community members, and supply distribution site staff and peers.

We endeavored to develop a simple survey tool for the collection of drug use data from HR clients, leveraging the existing supply distribution network to pilot the survey. The main objectives of this pilot survey were to identify substances commonly used by HR site clients in BC's five health regions and assess for geographic differences in drug use. A secondary objective was to compare the pilot survey data collected from various communities in each health region with an established cross-sectional survey which collects data in the major cities of Vancouver and Victoria.

## Methods

The 2012 survey tool was collaboratively designed with extensive input from provincial HR stakeholders through a series of working group meetings. An environmental scan was conducted to identify existing domestic and international surveys focusing on active drug use. Established drug survey tools were reviewed, and the working group prioritized the information to be collected and determined the survey format, question phrasing, and logistics of survey administration. The focus of the survey was current drug use among HR clients (i.e. the 7 days prior to participating in the survey) (Table 1).

**Table 1:** Survey summary

	**Description**	**Questions**
Page 1	Consisted of the following questions (paraphrased, multiple answer choices not provided)	Is this the first time you have used this supply pick up site?
		Gender?
		Age?
		Who are you picking up supplies for?
		Have you used the services of other supply pick-up sites?
		In the past 7 days, have you consumed alcohol not sold in a liquor store?
		If you used rigs in the past 7 days, how did you get rid of them?
Page 2	Asked about drugs used in the past 7 days^a^ (number of days used, method of use, doses(hits) per day, cost per hit)	Cocaine (powder)
		Crack
		Heroin
		Methadone
		Morphine
		Oxycontin/codone
		Dilaudid
		Amphetamines (dexedrine, preludin)
		Barbiturates
		Crystal meth
		‘Speedballs’
		Benzo's
		Ritalin
		Glues or solvents

Summary of the 2012 Survey Tool. ^a^Polysubstance use was defined as using ≥2 drugs from the list above and included other drugs not listed but identified in the final question; polysubstance use excluded marijuana and alcohol. Source: 2012 HR Drug Use Survey, BC Harm Reduction Strategies and Services.

**Figure 1:**
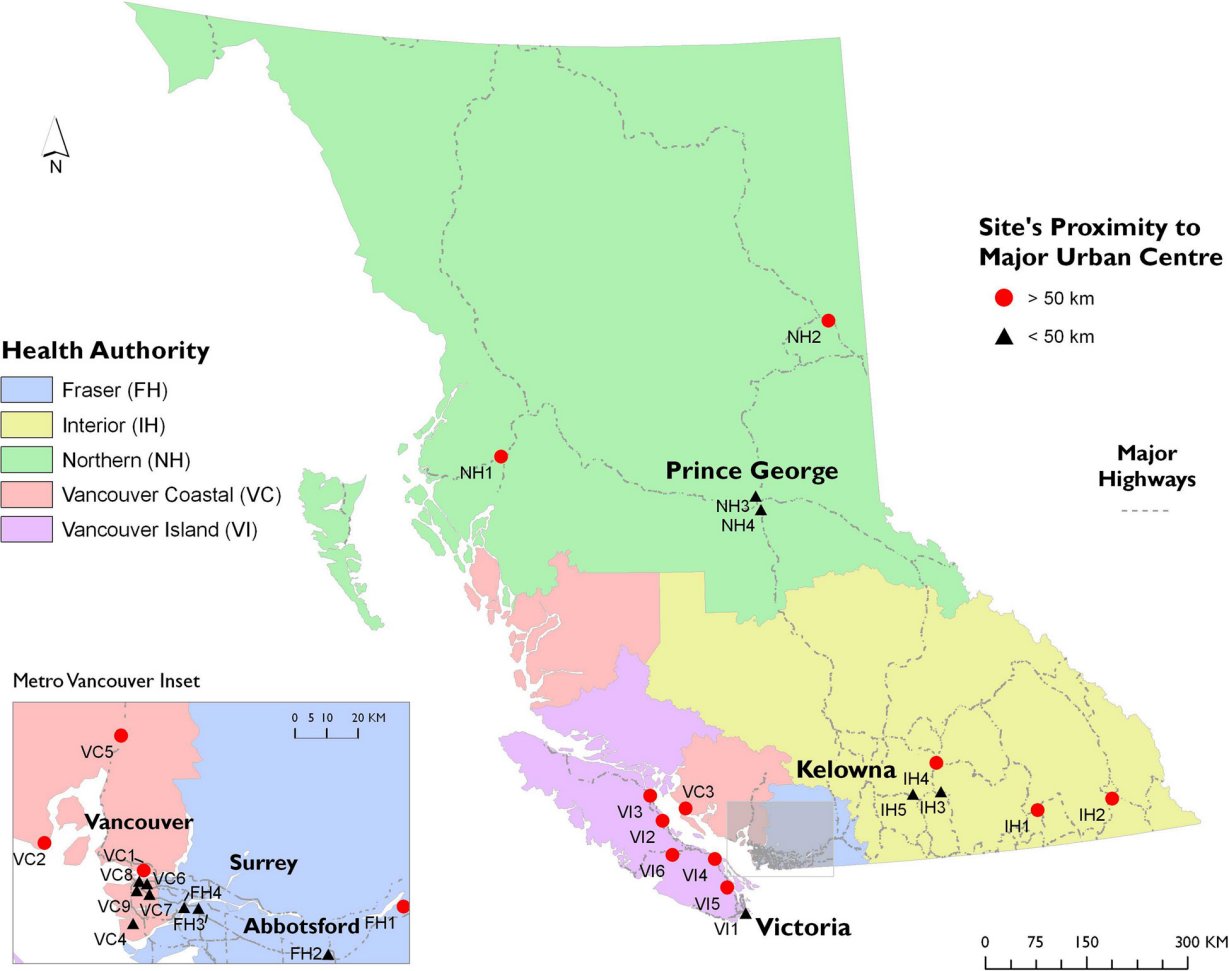
**Map of sites.** Harm reduction sites participating in Harm Reduction Client Drug Survey, 2012 (*N* = 28).

We utilized a two-stage convenience sampling method; sites were suggested by the regional HR coordinators based on capacity and willingness to participate. A total of 28 sites agreed to participate in the 2012 pilot survey, with four to six participating sites in each of the five regions (Figure 1). Site staff and peers then approached HR clients to invite participation.

The HR sites were contacted to discuss capacity and process for undertaking the survey. It was important that the survey be feasible for resource-limited HR sites to implement on a regular basis. *A priori*, pilot site staff were asked to identify criteria for feasibility for biannual or annual survey participation. From this, an acceptability questionnaire was developed to be administered to site groups after the survey was complete (Table 2). Additionally, staff and peers administering the survey to clients were contacted after the pilot to provide specific input on how individual questions were received by clients and feedback on how to improve the wording and administration of the survey. The process was open to all feedback on an ongoing basis during and after the survey period.

**Table 2:** Post-pilot survey summary

	**Description**	**Feedback**
Part 1	Consisted of 7 statements to be rated on a Likert scale:	1. We would be willing to do the survey again next year (once per year).
	☐ Strongly agree	2. We would consider doing the survey twice per year.
	☐ Agree	
	☐ Neutral	3. The questions were easy to understand and answer.
	☐ Disagree	
	☐ Strongly disagree	4. The staff and peers involved with the survey felt it was valuable information.
		5. The administration of the survey to clients was minimally disruptive to our regular activities.
		6. We estimate the decline/refusal rate was within acceptable limits (under 15%).
		7. Administration of the survey to clients by staff or peers is preferred over self-administration.
Part 2	Consisted of three open-ended statements for completion	1. What went well for us
		2. What did not go so well
		3. Suggestions for improvement
Part 3	Consisted of contacting select staff and peers who had administered the survey for feedback on wording of individual questions, how questions were received by clients, and suggestions for improvement	

Summary of 2012 post-pilot survey of staff and peers.

The survey materials were mailed out, and a 2-week period of data collection was designated. Each site received funding, $7 per survey, for client participation incentive and incidental costs, but the logistics of survey administration and handling of the participant incentives were determined at the site level. Most sites opted to provide participants $5–$7, while others provided the equivalent in coupons for meals or services. A few sites also used a portion of the funding to augment their regular provisions, such as fresh fruit or socks, for all who came in whether they participated or not. Peers are PWUD, currently or formerly, who assist service agencies with client engagement. Peer and staff interviewers were identified by the site lead and provided survey administration training materials. Training consisted of a pilot survey administration guide which provided the project framework, steps for obtaining verbal informed consent, and a script for phrasing each question.

HR clients were invited to participate if they were current clients of the site, 19 years or older, capable of giving verbal informed consent, had used any drugs (self-defined) in the prior 7 days, and had not participated in the survey previously. BC harm reduction sites are low-barrier environments in which clients are not asked for their names, age, or other personal information; government identification or healthcare cards; to exchange used needles/syringes to obtain clean ones; or, generally, to fill out forms. To maintain this approach, the survey was anonymous and no client information was linked to survey responses. Peers administering the survey reviewed the ethics-approved informed consent information with clients, confirmed understanding, and obtained verbal agreement as consent to participate, thus no signature was required. Participants were informed that they could decline any question and/or stop at any time and would not be asked about their decision nor would declining any part of the survey affect completing other questions, receiving the stipend, or receipt of any services.

The survey tool was two pages long and took approximately 10 min to administer by a peer or staff interviewer (Table 1). The first page collected basic demographics and information on HR site use, needle disposal practices, and consumption of non-beverage alcohol-containing products (alcohol not sold in a liquor store such as rubbing alcohol, hand sanitizer/microsan, mouthwash, cologne/after shave, hairspray, vanilla extract, cleaning products) [17]. The question on non-beverage alcohol was included due to stakeholder interest in assessing of crossover of the HR client site population with the illicit drinking population. The second page focused on type of drug(s) used in the past 7 days.

Using standard Canadian assessment tools which assess rurality/urbanicity, most BC communities are categorized as ‘urban’ due to the centralization of urban fringe and rural postal codes [18]. There are potential differences in drug distribution/availability between larger regional centres and those communities distant from larger centres. Further, most problem drug users, particularly the more marginalized, have limited financial means which may preclude travelling for longer durations or distances to obtain either drugs or HR supplies [19].

To explore possible differences in drug use, based on availability, between sites in the major regional centre and smaller outlying communities, we dichotomized HR sites according to distance from the health region's most populace city. We used a geographic information system, ArcGIS v.10.0 (ESRI, Redlands, CA, USA) to measure the distances from a point in the geographic centre of each major city to the geocoded points based on site addresses of that region. Using 50 km as the cutoff, we identified 13 sites ≤50 km and 15 sites >50 km from the health authorities major centre. An analytic decision was made to categorize two sites which mapped to within 50 km of the major centre as being outside of the major centre, as these sites almost exclusively served clients coming in from >50 km away from the city centre, based on site staff knowledge of client patronage.

Polysubstance use is a term with varying definitions [20]. We defined polysubstance use as having used two or more substances in the 7 days prior to the survey administration. For simplicity of questioning, we did not assess if use was simultaneous, within several hours, or days of each other. A list of common drugs was provided on the survey (Table 1, page 2), and the respondents were also asked about any other drugs they had used that did not appear on the list. Alcohol and marijuana were excluded from the list due to their presumed overall high prevalence and stakeholder interest at the time. We asked respondents to name all drugs they took in the prior 7-day period, regardless of whether or not there was a prescription, and did not record the presence or absence of a prescription for any drugs named.

CARBC provided substance use and prevalence data, also based on reported use in the past 7 days, from the 2012 Vancouver and Victoria surveys of street-involved adults. There were two data collection waves in 2012 in which the aim is to recruit 50 street-involved adults from both Vancouver and Victoria. In 2012, there were 257 CARBC survey participants asked about alcohol and drug use. Pearson chi-square was used to test for differences in drug use frequencies between the CARBC group and the pilot survey group for crack, heroin, morphine, cocaine, crystal methamphetamine, and amphetamines.

Survey data were entered into Microsoft® Office Access 2003. Data manipulation and statistical analyses were conducted in SAS® version 9.3. Graphs summarizing survey data were created in Microsoft® Excel 2003. All geocoding, spatial data analysis, and maps were conducted using ArcGIS v10.0 (ESRI, Redlands, CA, USA). The analysis focused on univariate and bivariate descriptive statistics of each question and reported drug frequencies; as well comparisons between health regions and by site/client proximity to a major centre. Chi-square tests were used for comparison of categorical data, and independent *t* tests were used for continuous data.

This project was approved by the University of British Columbia Behavioural Research Ethics Board and other relevant local boards.

## Results

**Figure 2:**
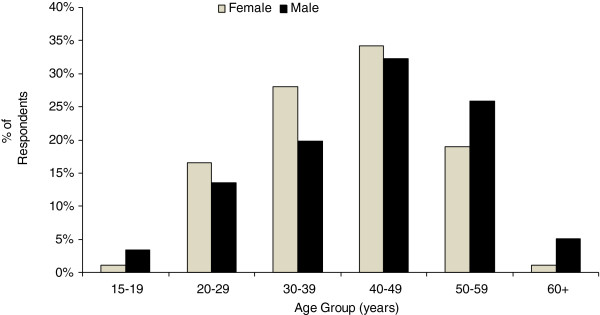
**Age-sex distribution.** Sex and age distribution of survey respondents, BC, 2012 (*N* = 739). One transgendered respondent and three respondents missing sex data were omitted from this figure.

There were 743 clients who completed the first page of the survey. Of these, 698 (93.9%) went on to complete the second page (drugs used in the past 7 days) (Table 3). Of the 743 respondents, 449 (60.8%) were male and 289 (39.1%) were female (1 transgendered; 3 sex missing). Males were older (*p* = 0.036), with a mean age of 42.6 years (range 19–74) compared to 40.0 years among females (range 19–80) (Figure 2).

**Table 3:** Number of respondents by region

	**Interior**	**Fraser**	**Vancouver coastal**	**Vancouver Island**	**Northern**	**Total**
Number of respondents completing page 1 (row%)	188 (25.3%)	163 (21.9%)	145 (19.5%)	129 (17.4%)	118 (15.9%)	743 (100%)
Number of respondents completing page 2 (row%)	178 (25.5%)	161 (23.1%)	141 (20.2%)	127 (18.2%)	91 (13.0%)	698 (100%)

Number of survey respondents completing pages 1 and 2 of the BC Harm Reduction Drug Use Survey, by health region, 2012 (*N* = 743).

Among respondents picking up harm reduction supplies from the site at the time of the survey, 42% were collecting supplies for themselves, 42% for themselves and others, 4% only for others, and 11% did not answer the question. Most respondents (76%) were repeat clients at the site where they were surveyed.

The majority of respondents (92%) reported that they had not consumed non-beverage alcohol, 6% reported they had, and 2% did not answer the question.

The level of safety of reported needle disposal methods among the 299 individuals who had disposed of needles in the prior 7-day period was positive with 78% categorized as safe (e.g. return to site, needle disposal container), 13% as somewhat safe (e.g. alternate closed container, garbage), and 9% as not safe (e.g. street, not sure).

**Figure 3:**
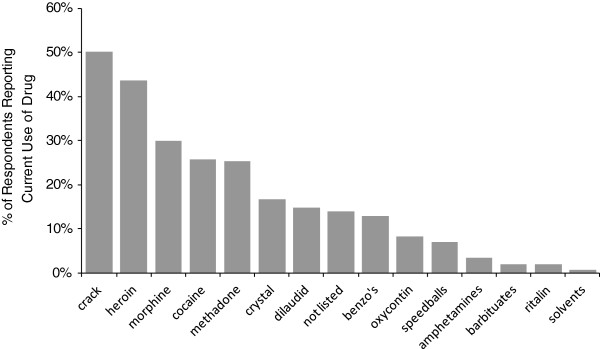
**Overall drug frequencies.** Percentage of respondents reporting drug use by drug type, BC, 2012 (*N* = 698).

The prevalence of drugs used in the prior 7-day period was compiled by type of drug, among all respondents and by health region. Crack cocaine use was reported by 50% of respondents; 44% reported heroin use, 30% had used morphine, and 26% had used cocaine (Figure 3).

The final survey question asked respondents if, in the prior 7 days, they had used any other drugs not listed in the survey. A key purpose of this question was to assess if any frequently taken drugs were excluded from the main list. As the final question, only a subset of respondents answered this question. Among those that responded to this question, marijuana and alcohol were most frequently reported but 23 persons reported a variety of other drugs such as diazepam, gabapentin, amitriptyline, acetaminophen, acetaminophen/codeine, seroquel, among others.

**Figure 4:**
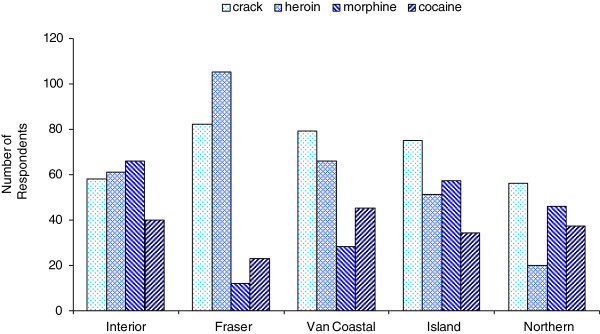
**Four drugs by health region.** Percentage of respondents reporting use of crack cocaine, heroin, morphine, and powder cocaine by health region, BC, 2012 (*N* = 698).

Crack was found to be the most common drug reported among respondents in three of the five regional health authorities (Figure 4)—Vancouver Coastal, Vancouver Island, and Northern Health Authorities. In the Interior health region, heroin and morphine use were slightly higher than crack use. A high proportion of Fraser respondents reported heroin use, followed by crack, with comparatively low levels of powder cocaine and morphine use. Northern respondents had the highest proportion of morphine use, and this was the only health region in which powder cocaine use was higher than heroin use.

**Figure 5:**
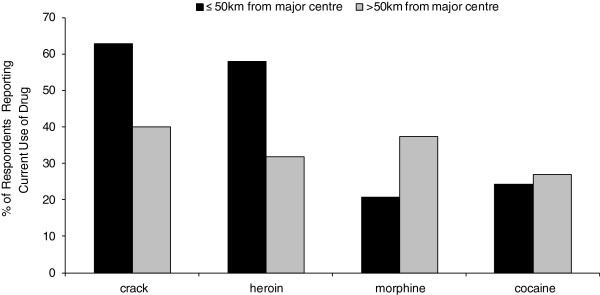
**Four drugs by distance from the major centre.** Percentage of respondents reporting use of crack cocaine, heroin, morphine, and powder cocaine by distance from the major centre, BC, 2012 (*N* = 698).

Drug use among respondents within 50 km of the major centre differed from those 50 km or more away (Figure 5). There was more crack and heroin use among respondents of sites in or near major centres (*p* < 0.0001 and *p* < 0.0001, respectively). Morphine use was found to be significantly greater among respondents accessing harm reduction at >50 km away from a major centre (*p* < 0.0001). There was no significant difference in the reported use of powder cocaine between the two defined populations (*p* = 0.4708).

**Figure 6:**
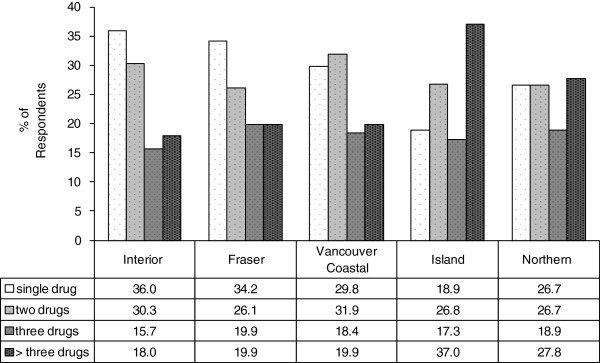
**Polysubstance use.** Polysubstance use among survey respondents by health region, BC, 2012 (*N* = 698). Polysubstance use was defined as using ≥2 listed drugs (marijuana and alcohol excluded).

Polysubstance use was common among HR clients surveyed in all health authorities (mean 69.9%; range 64.0%–81.1%) (Figure 6). Vancouver Island had the highest prevalence of polysubstance use (81.1%) and a notably high prevalence of reporting of greater than three substances used in the last 7 days (37.0%). Northern had the second highest prevalence of more than three substances used within last 7 days (27.8%). Among the 304 respondents who reported use of heroin in the prior 7-day period, 84% also used another substance in the same 7-day period: 54% reported use of crack, 31% reported use of methadone, and 24% and 23% reported use of cocaine and morphine, respectively.

We compared the findings of the 2012 CARBC High Risk Drug Survey among street-involved adults to our survey findings. Among HR clients responding to this survey, the prevalence of crack use was lower (*p* = 0.0004), while both heroin and morphine use was higher (*p* < 0.0001; *p* < 0.0001, respectively) than the CARBC survey respondents in Vancouver and Victoria (Table 4).

**Table 4:** CARBC comparison

	**2012 CARBC drug survey (*N*** **= 257) last 7 days (%)**	**2012 HR drug survey (*N*** **= 698) last 7 days (%)**	***p *** **value**
Crack	63.04	50.14	0.0004
Heroin	21.40	43.55	<0.0001
Morphine	14.01	29.80	<0.0001
Cocaine	21.40	25.64	0.1761
Crystal meth	18.29	16.62	0.5430
Amphetamine	3.11	3.30	0.8875

Percentage of respondents reporting use of crack cocaine, heroin, morphine, and powder cocaine by distance from the major centre, BC, 2012 (*N* = 698). CARBC, Centre for Addictions Research of BC; HR, harm reduction.

**Figure 7:**
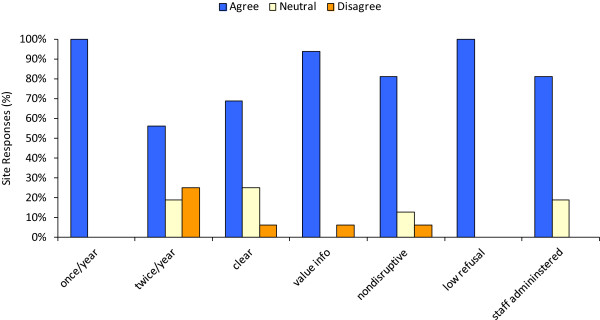
**Post-pilot survey.** Post-pilot survey responses BC, (*N* = 28 HR sites). See Figure 3 for complete statements and Likert scale.

In the post-survey questionnaire (Figure 7), pilot sites were in agreement with running the survey once each year, but fewer felt that it was feasible twice per year. Approximately 70% of the sites found the survey wording of questions clear; those sites that did not find the wording of all questions clear were asked for further input to improve clarity. Over 90% of the sites stated that they valued the information covered by the survey questions for their own knowledge and planning. Over 80% of the sites found the process non-disruptive, while others provided input into making the process less disruptive. Sites with low supply distribution volumes and staff numbers generally found it more challenging to administer the survey, but this also depended on the service model. Subjective refusal and non-completion levels were universally felt to be very low and acceptable, and this was attributed to the stipend offered to clients for their participation. Over 80% of sites reported that survey administration by site staff or peers (versus self-administration by clients) was necessary for both data quality and to maintain other benefits such as rapport building with clients.

Many sites reported that the survey process offered an opportunity for engagement and rapport building with clients as well as a way for staff to learn more about what is going on at street level and to identify areas of need. The anonymous nature of the survey and administration by peers was reported to be important to encourage participation and accurate responses. Site staff reported that the survey asked timely and relevant questions and can be used to ask questions of local interest.

However, there were challenges in administering the survey in winter (January/February) in Northern when client volumes were low (stocking up on supplies is more common when travel is restricted) and in sites with a small number of staff such that leveraging of students and volunteers was required. Sites reported that asking clients to estimate the amount of drug used ‘per hit’ for each drug reported was problematic.

Suggestions for improvement included the following: placing alcohol and cannabis on the main drug list in order to assess risk behaviours around combining particular drugs with alcohol; assessing simultaneous use of alcohol (i.e. taken at the same time or within several hours) with particular drugs, such as opiates; and asking about OD witnessing and experiences. It was felt that the ongoing use of peers is important to continue to improve clarity of the wording of survey. Finally, site staff and peers suggested that a video on survey administration would be an effective training tool.

## Discussion

This survey provided data on drug use in a sample of harm reduction clients who actively use drugs from each of BC's five geographic health regions using a simple survey tool. Differences in the most common drugs used and levels of polysubstance use were found between sites and health regions, and based on community distance to the regional centre. With further development, this survey approach may inform HR stakeholders about client drug use with the aim of optimizing regional and local services for the prevention of drug-related harms. That drug use differed substantially by sites, regions, and community type is an important finding underlining the need for locally collected data to inform service planning. While the survey is not community-based, the tool may be customized to collect information on topical local or regional issues.

Overall, crack was the most commonly reported drug used, followed by heroin then morphine.

Canadian cohort data has long indicated major increases in crack use over time and that crack is now the most prevalent drug used in many major metropolitan areas [6,21]. We also found that respondents from sites in or near the major centre had a higher prevalence of crack use. Many studies have reported that persons who use crack are among the most socio-economically marginalized groups and are associated with high rates of homelessness, polysubstance use, and HIV and/or HCV co-infection [21-24]. However, compared with CARBC data from BC's two major cities, we found a lower prevalence of crack use which may be due, in part, to recruitment from HR sites where more injection supplies than crack smoking supplies are available.

Fischer et al. report that there are fewer established supports in BC for persons who smoke crack as compared to persons with opioid dependence or injection drug use [22]. We found >50% prevalence of crack smoking among harm reduction clients surveyed. Currently, the BC Harm Reduction Program does not provide glass stems (crack pipes) for safer crack use in their supply distribution (though some health regions support distribution at specific local sites) nor have safe inhalation sites been evaluated for implementation in BC [25,26].

Another proposed risk reduction measure for persons who smoke crack is the co-use of cannabis in order to manage psycho-stimulant effects [27]. Further iterations of the survey will include questions to evaluate access to glass stems (sharing, use of makeshift pipes), access to safe locations for illicit smoking, and the prevalence of co-use of cannabis as a harm reduction measure.

Northern was the only health region in which the reported use of morphine was higher than that of heroin. There was more heroin use among respondents at sites closer to the major centre versus more morphine use at those further away. These finding are in keeping with evidence that prescription opioids diverted for illicit use is a growing problem in Canada [4-6]. Fischer et al. found evidence that heroin has become an increasingly marginal drug used among illicit opioid users in Canada, particularly outside of major port cities thought to be heroin import points, such as Montreal and Vancouver [4]. To better understand the level of diversion of prescription opioids, future surveys will ask about whether or not clients had a prescription for drugs reported.

We identified key gaps in our survey to be addressed in future iterations. These included the inability to assess (1) alcohol and marijuana use, (2) the concurrent use of drugs, and (3) overdose. In 2006, Buxton et al. assessed a year of coroner's data on drug overdose deaths in BC [28]. In 34.5% of cases, there were three or more substances detected. In our study, 41.4% of the overall sample reported three or more substances used in the prior 7 days, although our definition excluded alcohol and marijuana. Among overdose deaths in 2006, cocaine was identified in 80.3% and opiates in 59.6% of deaths, with opioids more frequently identified in Vancouver (74.1%) compared to outside Vancouver (55.0%). Both morphine and cocaine were detected in 44.4% of deaths (55.3% of cocaine-positive cases). Alcohol was detected in 22.6% opiate-positive cases. As combining alcohol with opiates plays a clear role in respiratory depression, including alcohol is important in assessing overdose risk and educational needs among harm reduction clients. Understanding polysubstance use trends is important to planning BC's public health interventions for overdose.

Reported polysubstance use (≥2 substances) was highest in Northern and Vancouver Island health regions. The Northern health region is remotely located from Vancouver, as BC's port city, and Vancouver Island health region is separated from the mainland, requiring ferry transport. This could potentially affect the availability of particular drugs such as heroin [2-4]. Identifying areas of high opioid and polysubstance use allows strategic rollout of overdose prevention initiatives such as BC's recently initiated Take Home Naloxone Program [29].

This first survey implementation and evaluation have provided valuable refinements to future data collection, including (1) adding alcohol and marijuana to the drug list, (2) specifically asking about concurrent drug combinations (within 6 h of each other), and (3) adding specific questions on client residence/distance travelled to site, sharing needles, drug overdose, prescriptions, and crack pipe access.

For practicality, we used a small convenience sample to pilot this survey. Due to site self-selection, the low number of sites per health region, and mixed urbanity/rurality of the sites, the collated data from all participating sites in a single health region cannot be generalized to the region as a whole. The goal of future surveys is to expand to more sites throughout the province, which will improve regional representativeness. However, each site can benefit from receiving the summary of its own data. Going forward, data aggregated at the regional level may contribute to major initiatives coordinated at the regional level, and local data will be required for local service planning.

Survey findings are limited to harm reduction clients and thus may not represent PWUD who do not access harm reduction sites (e.g. mainstream youth or who have supplies picked up by a friend). As we are asking clients about their drug use in the recent past, recall bias and social acceptability bias may play roles in underreporting of certain drug use.

## Conclusion

Pilot survey data showed differences in drug use among harm reduction clients by region and by proximity to a major centre. That drug use differed substantially by sites, regions, and community type is an important finding underlining the need for locally collected data to inform service planning. Differences in drug use trends found between this survey and established surveys further the hypothesis that information on drug use from regions outside of the major metropolitan regions is of value in planning prevention activities in other regions of the province.

## Abbreviations

BC: British Columbia; CARBC: Centre for Addictions Research of British Columbia; HCV: Hepatitis C virus; HIV: Human immunodeficiency virus; HR: Harm reduction; PWID: Persons who inject drugs; PWUD: Persons who use drugs; THN: Take-home naloxone.

## Competing interests

The authors declare that they have no competing interests.

## Authors' contributions

MK conducted the survey, analysed the results, prepared all tables and figures (except the map), prepared a stakeholder report, and finalized the manuscript. AS wrote the first draft of the manuscript and coordinated input sessions and early revisions. DT participated in data analysis and provided input into the developing drafts. JB was the project lead and participated in data analysis decisions and direction of the developing drafts. All authors read and approved the final manuscript.

## Authors' information

JB is the chair of BC's Harm Reduction Strategies and Services (HRSS) committee and developer and lead of BC's THN Program. DT was the HR coordinator involved in the rollout of the BC's THN Program.

## References

[B1] Degenhardt L Hall W Extent of illicit drug use and dependence, and their contribution to the global burden of disease Lancet 2012 379 9810 55 70 10.1016/S0140-6736(11)61138-0 22225671

[B2] Ciccarone D Heroin in brown, black and white: structural factors and medical consequences in the US heroin market Int J Drug Pol 2009 20 3 277 282 10.1016/j.drugpo.2008.08.003 PMC2704563 18945606

[B3] Wood E Stoltz JA Li K Montaner JSG Kerr T Changes in Canadian heroin supply coinciding with the Australian heroin shortage Addiction 2006 101 5 689 695 10.1111/j.1360-0443.2006.01385.x 16669902

[B4] Fischer B Rehm J Patra J Firestone CM Changes in illicit opioid use across Canada CMAJ 2006 175 11 1385 1387 10.1503/cmaj.060729 17116905 PMC1635767

[B5] Gfroerer JC Larson SL Colliver JD Drug use patterns and trends in rural communities J Rural Health 2007 23 s1 10 15 10.1111/j.1748-0361.2007.00118.x 18237319

[B6] Haydon E Fischer B Crack use as a public health problem in Canada: call for an evaluation of ‘safer crack use kits’ Can J Public Health 2005 96 185 188 15913081 10.1007/BF03403687 PMC6976094

[B7] Collier R Street versions of opioids more potent and dangerous CMAJ 2013 185 12 1027 10.1503/cmaj.109-4535 23836854 PMC3761004

[B8] BC Centre for Excellence in HIV/AIDS Vancouver Injection Drug Users Study, (VIDUS) [cited March 14, 2014]; Available from: http://cfenet.ubc.ca/research/vidus/

[B9] BC Centre for Excellence in HIV/AIDS Urban Health Research Initiative. At-Risk Youth Study (ARYS) [cited March 14, 2014]; Available from: http://cfenet.ubc.ca/research/arys/

[B10] Yang J Oviedo-Joekes E Christian KWM Li K Louie M Schechter M Spittal P The Cedar Project: methadone maintenance treatment among young Aboriginal people who use opioids in two Canadian cities Drug Alcohol Rev 2011 30 6 645 651 10.1111/j.1465-3362.2010.00258.x 21355933

[B11] University of Victoria, Centre for Addiction Research of BC (CARBC) Alcohol and Other Drugs Monitoring Project (AOD Project). High risk populations [cited March 14, 2014]; Available from: http://www.carbc.ca/AODMonitoring/ProjectComponents/HighRiskPopulations/tabid/89/LiveAccId/7533/Default.aspx

[B12] Public Health Agency of Canada Chapter 3: HIV/AIDS Epi updates - HIV testing and surveillance systems in Canada 2010 [cited March 14, 2014]; Available from: http://www.phac-aspc.gc.ca/aids-sida/publication/epi/2010/3-eng.php

[B13] Centers for Disease Control and Prevention Updated guidelines for evaluating public health surveillance systems: recommendations from the guidelines working group MMWR 2001 50 No. RR-13 1 35 18634202

[B14] Fielden SJ Marsh DC It's time for Canadian community early warning systems for illicit drug overdoses Harm Reduction J 2007 4 10 1 15 10.1186/1477-7517-4-10 PMC1851954 17391529

[B15] BC Harm Reduction Strategies and Services Committee BC Harm Reduction Strategies and Services Committee policy indicators report 2013 [cited March 30, 2014]; Available from: http://www.bccdc.ca/NR/rdonlyres/B39C410C-F5D1-467B-A92F-B46715583404/0/BCHRSS2011PolicyIndicatorsReportFINAL.pdf

[B16] BC Harm Reduction Program Find a harm reduction site (interactive map) [cited March 14, 2014]; Available from: https://towardtheheart.com/site-locator

[B17] Stockwell T Vallance K Martin G Macdonald S Ivsins A Chow C Greer A Zhao J Duff C Lucas P March D Michelow W Treno A The price of getting high, stoned and drunk in BC: a comparison of minimum prices for alcohol and other psychoactive substances 2010 [cited March 14, 2014]; Available from: http://www.carbc.ca/portals/0/propertyagent/558/files/22/carbcbulletin7.pdf

[B18] Statistics Canada Rural and Small Town Canada Analysis Bulletin: definitions of rural 2001 [cited March 14, 2014]; Available from: http://www.statcan.gc.ca/pub/21-006-x/21-006-x2001003-eng.pdf

[B19] Gyarmathy VA Neaigus A Marginalized and socially integrated groups of IDUs in Hungary: potential bridges of HIV infection J Urban Health 2005 82 3 Suppl 4 iv101 iv112 16107433 10.1093/jurban/jti112 PMC2656943

[B20] Olthuis JV Darredeau C Barrett SP Substance use initiation: the role of simultaneous polysubstance use Drug Alcohol Rev 2013 32 1 67 71 10.1111/j.1465-3362.2012.00470.x 22612987

[B21] Fischer B Santos Cruz M Bastos FI Tyndall M Crack across the Americas – a massive problem in continued search of viable answers: exemplary views from the North (Canada) and the South (Brazil) Int J Drug Pol 2013 24 6 631 633 http://dx.doi.org/10.1016/j.drugpo.2013.09.003 10.1016/j.drugpo.2013.09.003 24120442

[B22] Fischer B Rehm J Patra J Kalousek K Haydon E Tyndall M El-Guebaly N Crack across Canada: comparing crack and non-crack users in a multi-city cohort of opioid and other street drug users Addiction 2006 101 12 1760 1770 10.1111/j.1360-0443.2006.01614.x 17156175

[B23] Fischer B Rudzinski K Ivsins A Gallupe O Patra J Krajden M Social health and drug use characteristics of primary crack users in mid-sized communities in British Columbia, Canada Drugs: Educ, Prev, Pol 2010 17 4 1340 1341

[B24] DeBeck K Kerr T Li K Fischer B Buxton JA Montaner J Wood E Smoking of crack cocaine as a risk factor for HIV infection among people who use injection drugs CMAJ 2010 181 9 585 589 19841052 10.1503/cmaj.082054 PMC2764753

[B25] DeBeck K Buxton JA Kerr T Qu J Montaner J Wood E Public crack cocaine smoking and willingness to use a supervised inhalation facility: implications for street disorder Subst Abuse Treat Prev Pol 2011 6 4 10.1186/1747-597X-6-4 PMC3049126 21345231

[B26] Fischer B Tiesmaki M Rudzinski K Lusted A Effectiveness of Secondary Prevention and Treatment Interventions for Crack Use in English-Language Jurisdictions: A Narrative Review 2010 Brazil: Brazilian Ministry of Health & the Pan-American Health Organization

[B27] Andrade T Santiago L Amari E Fischer B ‘What a pity!’ – exploring the use of ‘pitilho’ as harm reduction among crack users in Salvador, Brazil Drugs: Educ, Prev, Pol 2011 47 13–14 382 386

[B28] Buxton JA Skutezky T Tu AW Waheed B Wallace A Mak S The context of illicit drug overdose deaths in British Columbia, 2006 Harm Reduction J 2009 6 9 http://www.biomedcentral.com/content/pdf/1477-7517-6-9.pdf 10.1186/1477-7517-6-9 PMC2694163 19480677

[B29] BC Harm Reduction Program Naloxone Program Available at: https://towardtheheart.com/naloxone/

